# Beyond the here and now: hunter–gatherer socio-spatial complexity and the evolution of language

**DOI:** 10.1098/rstb.2022.0521

**Published:** 2024-09-04

**Authors:** Brian M. Wood, David A. Raichlen, Herman Pontzer, Jacob A. Harris, M. Katherine Sayre, Bunga Paolo, Mariamu Anyawire, Audax Z. P. Mabulla

**Affiliations:** ^1^ Department of Anthropology, University of California, Los Angeles, CA, USA; ^2^ Department of Human Behavior, Ecology and Culture, Max Planck Institute for Evolutionary Anthropology, Leipzig, Germany; ^3^ Department of Biological Sciences and Anthropology, University of Southern California, Los Angeles, USA; ^4^ Department of Evolutionary Anthropology, Duke University, Durham, NC, USA; ^5^ Duke Global Health Institute, Duke University, Durham, NC, USA; ^6^ School of Interdisciplinary Forensics, Arizona State University, Tempe, AZ, USA; ^7^ Department of Anthropology, University of California, Santa Barbara, CA, USA; ^8^ Hadza fund, Mangola, Tanzania; ^9^ Department of Archaeology and Heritage, University of Dar es Salaam, Dar es Salaam, Tanzania

**Keywords:** hunter–gatherers, human movement ecology, displacement, Hadza, olive baboon, evolution of language

## Abstract

Human evolutionary ecology stands to benefit by integrating theory and methods developed in movement ecology, and in turn, to make contributions to the broader field of movement ecology by leveraging our species’ distinct attributes. In this paper, we review data and evolutionary models suggesting that major changes in socio-spatial behaviour accompanied the evolution of language. To illustrate and explore these issues, we present a comparison of GPS measures of the socio-spatial behaviour of Hadza hunter–gatherers of northern Tanzania to those of olive baboons (*Papio anubis*), a comparatively small-brained primate that is also savanna-adapted. While standard spatial metrics show modest differences, measures of spatial diversity, landscape exploration and spatiotemporal displacement between individuals differ markedly. Groups of Hadza foragers rapidly accumulate a vast, diverse knowledge pool about places and things over the horizon, contrasting with the baboon’s narrower and more homogeneous pool of ecological information. The larger and more complex socio-spatial world illustrated by the Hadza is one where heightened cognitive abilities for spatial and episodic memory, navigation, perspective taking and communication about things beyond the here and now all have clear value.

This article is part of the theme issue ‘The spatial–social interface: a theoretical and empirical integration’.

## 1. Introduction

Evolutionary anthropologists, linguists and cognitive scientists have suggested that changes in socio-spatial behaviour accompanied the evolution of key attributes of *Homo sapiens*, including our large brains, advanced cognitive capabilities and language [1–3]. These evolutionary models motivate research into functional relationships between spatial behaviour, social structure, cognition and communication, aligned with the socio-spatial interface framework advocated by Webber *et al*. [4]. Humans and our hominin ancestors are extreme outliers in our dependence on socially transmitted information [5]. Following the causal paths outlined by Webber *et al*. [4], we thus expect distinct patterns of spatial behaviour to have emerged in our species, relative to other primates. Specifically, we hypothesize that patterns of spatial behaviour have arisen that facilitate increased collection and sharing of ecological information. To examine this idea, Part 1 of this paper presents a targeted review of relevant theory and data. In Part 2, we provide an empirical comparison, using GPS data collected at a similar scale and resolution, of the movement and interaction patterns of human hunter–gatherers and olive baboons living in a similar savanna–woodland habitat.

### (a) Part I: the evolution of human spatial behaviour and language

Reconstructing the evolution of hominin spatial behaviour is challenging because data attesting to ancestral human landscape use is scant and fragmentary. Tentative models of the past are built through a merging of theory with data derived from studies of primatology, fossil anatomy, geology, archaeology and ethnography. Primatological research documents the range of challenges that early hominins likely confronted, inspiring hypotheses about how adaptations to these challenges shaped human evolution. Studies of great apes are particularly relevant for phylogenetic reconstructions of early hominins.

Chimpanzees, bonobos and gorillas all specialize in acquiring ripe fruit and prefer rainforest habitats with short dry seasons [6]. The last common ancestor of humans and African apes likely had similar dietary and habitat preferences [7]. Since the Late Miocene, global cooling, drying and increased seasonality have led to the spread of grasslands and open woodlands across much of Africa [8]. Stable carbon isotopes from fossil soils indicate that over the past 6 million years, hominins in eastern Africa lived in open environments with less than 40% woody cover [9].

Remains of early hominins in the period between 7 and 4 Mya have been found in central and east Africa, and have been placed within the genera *Sahelanthropus*, *Orrorin* and *Ardipithecus* [10,11]. Anatomical interpretations of these earliest hominins remain a focus of debate, but they appear to have been bipedal, with the skulls of *Sahelanthropus* and *Ardipithecus* indicating great ape-sized brains [12,13]. Unambiguous evidence for bipedalism is found in the anatomy of *Australopithecus* species after 4 Mya [14]. When modelling how these early hominins made a living and ranged through landscapes, large primates living in open environments are especially relevant. In a less fruit-rich environment, one strategy is to consume large amounts of lower-quality vegetation. When facing seasonal fruit shortfalls, mountain gorillas, for instance, focus on abundant but low-quality fibrous leaves and terrestrial herbaceous vegetation (THV) and maintain smaller daily travel distances than other apes [15–18]. A similar strategy might have been adopted by the megadont hominin *Paranthropus* group (3–1 Mya), which likely exploited low-quality C4 vegetation [19,20].

Another strategy in response to decreased fruit abundance is to expand one’s search for higher quality foods. Evidence for an analogous strategy is seen in comparisons of chimpanzee communities. Compared with those living in fruit-rich rainforests, chimpanzees at the far western and eastern edges of the species’ range, in the savanna woodlands of Senegal and Tanzania (Mt. Assarik, Fongoli and Ugalla, Tanzania), cover more territory and have the largest home ranges recorded for any non-human primate [21,22].

Baboons are also a widely used primate model for early hominins [23,24]. They are an apt model for considering the ranging and sociality of australopithecines because both taxa are large-bodied, sexually dimorphic, catarrhine primates [25]. Olive baboons are ecologically diverse and widespread, inhabiting both forest and savanna habitats across Africa. Many *Australopithecus* remains have been recovered from the East African Rift Valley [26], a region where today baboons thrive, and no great ape populations are found. For thinking about how the risks, resources and physical features of this habitat would have influenced the spatial and social behaviour of australopithecines, further consideration of the baboon model is warranted. Comparing the spatial behaviour of humans and baboons today may provide insights into how our species’ unique cognitive abilities, technologies and language have changed the manner in which we make adaptive use of these landscapes, relative to a more primitive ancestral species.

Language is our species’ most distinctive adaptation [27]. A core feature of language is that it permits efficient communication about spatially and temporally remote events beyond the here and now [28–30]. The linguist Charles Hockett [30] argued that an exceptional feature of human language was its capacity for *displacement*—the ability to communicate about things that are out of sight or in the past or future. By contrast, signals used in non-human communication systems typically refer to things in the immediate environment of the sender or receiver, such as the presence of a predator or a food source. Hockett considered some capacity for displaced communication to be a key precursor to the development of more complex symbolic communication. More recently, linguist Derek Bickerton [29] highlighted the extreme rarity of displacement in animal communication systems. He argued that this feature was pivotal in the early evolution of human language. Bickerton proposed that the capacity for displacement likely evolved for sharing information about food sources located beyond the direct sensory perception of message recipients. Displacement is the feature of language we focus on here because it refers to the *where* and *when* of meanings being expressed in language, and the adaptive benefits of this rare, essential language feature hinge on how individuals interact across space and time. We hypothesize that this core feature of language is necessary only in social groups where individuals interact with others who have recently acquired dissimilar ecological and spatial information. Below, we test the prediction that human hunter–gatherer movement patterns more strongly satisfy this criterion than those observed in olive baboons.

### (b) Part 2: human hunter–gatherer and olive baboon movement patterns

Motivated by an interest in spatial information acquisition and sharing, we use GPS measures to estimate the spatial information that individuals are exposed to. Strandburg-Peshkin *et al.* [31] deployed GPS collars and tracked the movement of a troop of olive baboons at Kenya’s Mpala field station over 14 days, and then shared the GPS data publicly via moveBank [32]. These baboon movement data now provide a valuable benchmark for cross-species and cross-community comparisons. No such data are available for any great ape. The Hadza hunter–gatherer GPS data that we analyse here is the same sample used in Wood *et al*. [33], which records the movement of 179 Hadza research participants (87 females and 92 males) over 2048 person-days of travel, ranging in age from 2 to 84 years (mean = 36, s.d. = 19). We refer readers to that open-access publication for full details of the sample and methods. In studies of people, ethical considerations limit the open distribution of detailed location data, highlighting the importance of quantitative, standardized data summaries in order to balance the goals of knowledge dissemination with privacy and ethical concerns.

## 2. Results

We next compare measures of daily travel, inter-individual proximity, inter-individual differences in exposure to spatial information and collective measures of landscape exploration.

### (a) Daily travel and inter-individual proximity

**Figure 1.**
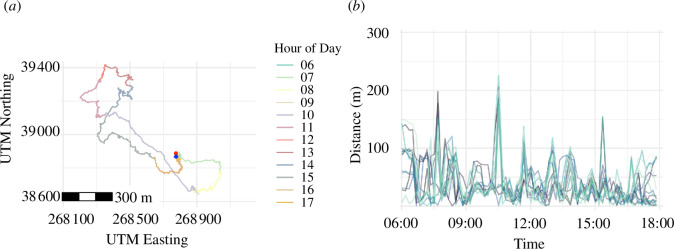
(*a*) The path of an adult female olive baboon at Mpala who travelled 10.6 km on 12 August 2012, over the course of 12 h. Her path on this day began at the location indicated by the red dot and ended at that shown by the blue dot, only 20 m distant. (*b*) Proximity between this female and all other group members throughout the day, sampled at 10-min intervals for visual clarity. Each line in (*b*) represents her proximity to a different group member.

The average day range for the Mpala baboons was 10.58 km (*n* = 250, s.d. = 1.81), higher than the day ranges reported for this species in prior research [34], likely owing to the high temporal resolution of the GPS data. This value is quite similar to the average day range of adult Hadza, which is 11 km on average for adults (males and females combined) between the ages of 18 and 50 [33]. In figure 1, we plot the track of the baboon whose day range most closely matched the sample average and her proximity to other group members throughout the day.

The Mpala baboons were central place foragers whose travel was usually tethered to the sleeping sites from which they departed in the morning and returned at the end of the day. Across the baboon sample, the median distance between a day’s start and end points of travel was only 58 m (s.d. = 85). Olive baboon sleeping sites are usually carefully selected for their safety and proximity to water and are often on cliffs or high in trees, where group members are afforded greater visibility and safety from predators [35].

**Figure 2.**
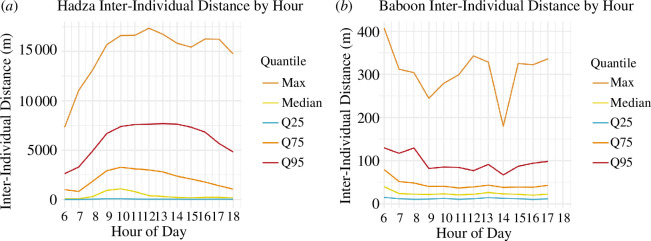
(*a*) Hadza inter-individual proximities by hour (*n* = 102 854 437) and (*b*) Mpala baboon inter-individual proximities by hour (*n* = 18 881 268). Please note the large difference in *y*-axis scales.

In an early study of wild baboons, Washburn & DeVore [36] wrote that ‘whether by day or night, individual baboons do not wander away from the troop, even for a few hours ... once an animal is separated from the troop the chances of death are high’ (ibid: 66). Indeed, the Mpala baboons seldom ventured far from one another (figure 1*b*). In the baboon sample, the median inter-individual distance was 23.1 m (mean = 33.5, s.d. = 32.9, max = 407.2, *n* = 18 881 268). Among the Hadza, the median inter-individual distance was 301.5 m (figure 2*a*; mean = 1545.8, s.d. = 2341.2, max = 17 347.3, *n* = 106 429 818).

In Hadza settlements, or camps, it is normal for people to depart camp in the early morning, travelling in parties of variable sizes, searching for and harvesting resources, followed by a return to camp in the afternoon or evening. A small subset of camp members might stay in the bush hunting all night, or leave on short visits to neighbouring camps [37]. These typical patterns of movement mean that the splitting and merging of Hadza social groups, reflected in inter-individual proximities, are highly structured across time, forming a general pattern of early day aggregation, daytime dispersal and evening re-aggregation. This forms a notable contrast with baboons, where cohesive groups are observed across all hours of the day (figure 2*b*); there is even a trend seen in the baboon GPS data of greater dispersion at dawn and in the evening.

**Figure 3.**
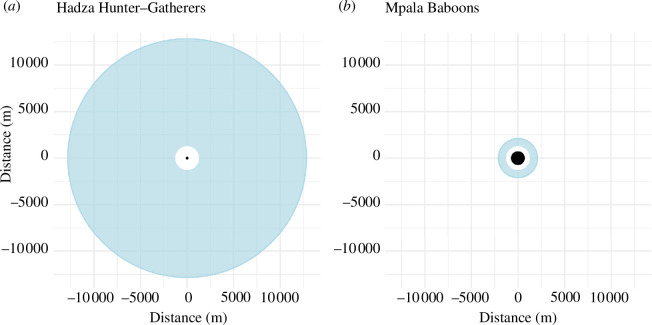
Time spent at different distances from each day’s starting point in the Hadza hunter–gatherer and Mpala baboon samples. The black circles in the centre of each plot encompass 50% of all GPS points, i.e. 50% of all recorded time, of each sample. The white circles encompass 75% of all GPS points, and the blue circles encompass 100% of each sample.

Hadza camps are inviting places where a range of activities occur including rest, food preparation, meals, childcare, socializing, play, tool maintenance, conversation and storytelling [37]. The importance of these in-camp social activities is reflected in the greater amount of time that Hadza spent very near their day’s first GPS point, which was usually at the wearer’s hearth (figure 3). Twenty-five per cent of the Hadza’s total GPS points (i.e. 25% of their total observed time) was within 24 m of their day’s starting point, and 50% of their total time was spent within 88 m of this point (figure 3).

By contrast, in the olive baboon sample, the first quartile of their recorded distances extends to locations 243 m distant from their day’s starting point, and the median logged distance was 679 m from that point. These data also attest to how much further the Hadza travel from their ‘central places’. No baboon travelled further than 2.1 km from their day’s starting point, whereas 17% of the Hadza’s time was spent in locations more distant than this, and the maximum distance reached by a Hadza in this sample was 12.8 km. In summary, these data show that Hadza both spend more time very close to their central place and more time very far from that place (figure 3).

### (b) Inter-individual differences in exposure to spatial information

**Figure 4.**
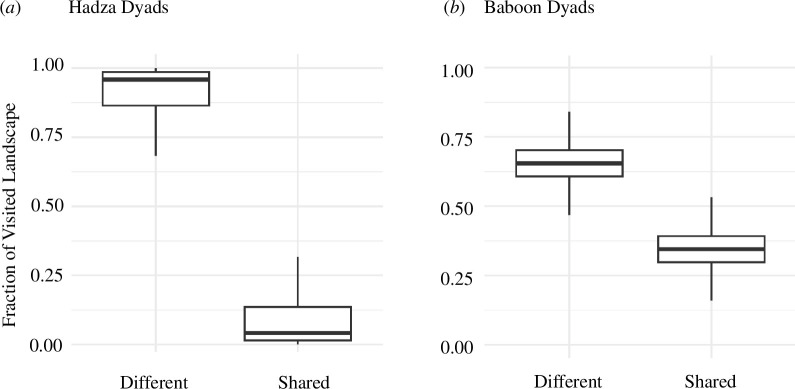
Dyadic measures of similarity and difference in daily landscape use by (*a*) Hadza hunter–gatherers (*n* = 13 656 dyads) and (*b*) Mpala baboons (*n* = 2240 dyads). See §4*d* for details of how these values were calculated.

Here we present measures of whether individuals explore the same or different parts of the landscape on each day, which we consider a proxy for differences in exposure to spatial and ecological information. These analyses are based on raster representations of the landscape, in which we record every 10 m^2^ cell visited by each individual, each day. For each dyad on each day, the fraction of their landscape use that is *shared* is that fraction of the total visited landscape that was visited by both individuals, and the fraction that is *different* is that proportion which was visited by one of the dyad members but not both. Our calculations show that dyads of Mpala olive baboons are much more similar in their patterns of landscape use and exposure to spatial information than are dyads of Hadza hunter–gatherers. The median shared fraction of the landscape visited per day was 0.35 in the Mpala baboon dyads and just 0.04 in the Hadza dyads (figure 4).

These data show that Hadza who live in the same camp experience much greater spatial displacement from one another each day (figure 2) and that dyads of Hadza who live and interact daily in the same group visit highly distinct areas of the landscape, relative to baboons (figure 4). This confirms our prediction that within Hadza groups, individuals would acquire more individually distinct (i.e. dissimilar) sets of spatial and ecological information. The Hadza’s spatial behaviour thus creates more possibilities for individuals to share information that is novel to others, because it was beyond others’ range of travel and direct sensory perception that day.

### (c) Collective measures of landscape exploration

We next consider how spatial exploration, and by extension, ecological and spatial information, accumulates in the group across individuals. We calculated the cumulative sum of the total unique land visited each day, progressively considering how this value changes as we sum from 1 to *N* individuals whose travel was monitored each day.

**Figure 5.**
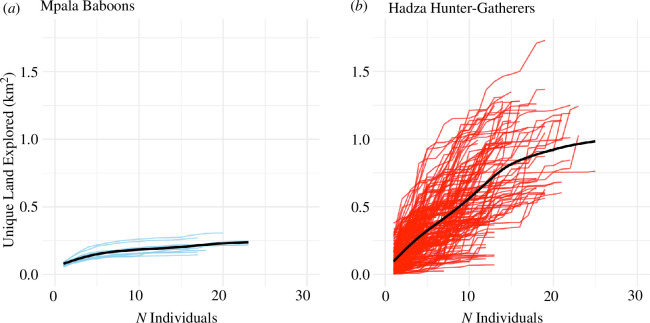
The cumulative amount of unique land explored per day, as a function of group size. Each line represents a unique calendar day, and its slope represents the relationship between the unique land explored and additional group members. The black line represents a generalized additive model of the expected trend, fit to the data using a cubic spline.

The cohesive ranging of baboons creates a relatively homogeneous spatial and ecological experience for group members. The low slope of the trend line in figure 5*a* means that additional group members provide little gain in spatial exposure, or by extension, unique spatial knowledge, at the collective level. Conversely, the Hadza’s more diverse travel results in a much steeper gain of exposure to unique areas of the landscape, producing a larger and more diverse pool of spatial information at the group level.

### (d) Sex differences in landscape use

A sexual division of labour, in which each gender is associated with different economic roles, is a universal feature of hunter–gatherer societies [38,39]. The sexual division of labour is one factor that helps explain why hunter–gatherers like the Hadza are expected to exhibit greater intra-group diversity in patterns of landscape use compared to non-human primates. Hadza men focus on hunting more widely dispersed and mobile resources (mammals, birds and wild bee colonies), whereas Hadza women focus on gathering immobile and more abundant plant resources (tubers, fruit and greens). A prior study of Hadza movement ecology [33] found that the paths that women followed while foraging out of camp were shorter and more linear, while those of Hadza men were longer and more sinuous. Here, we present a comparison of geographic segregation by sex/gender. Using raster analysis, we calculated the total landscape visited and then partitioned it into the fraction only visited by males, only visited by females, and that visited by both males and females.

**Figure 6.**
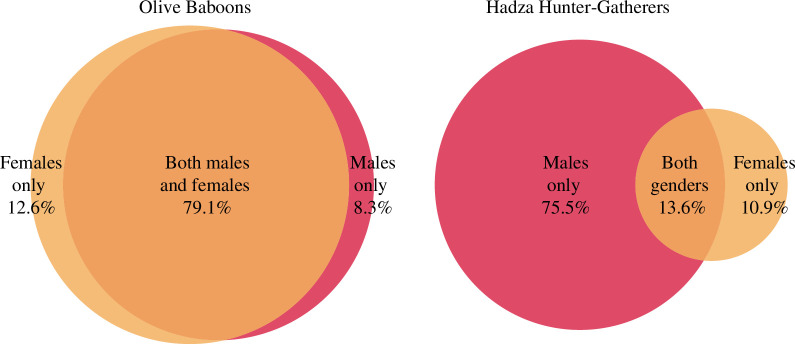
Similarity and difference in landscape use, aggregated by gender/sex. The Hadza measures are the average values in a study of 12 camps [33].

Figure 6 shows that gender strongly structures patterns of Hadza landscape use, and on average only 13.6% of the total landscape visited was visited by both males and females. This pattern arises because Hadza men travelled further, and usually travelled alone, whereas Hadza women travelled in groups and to fewer areas of the landscape [33]. Sex is much less predictive of landscape use in baboons, and both males and females visited 79.1% of the total visited landscape at Mpala.

## 3. Discussion and conclusion

While both Hadza hunter–gatherers and Mpala baboons inhabit similar environments in the Rift Valley and engage in central place foraging with similar daily travel distances, notable differences exist in their social interactions, travel diversity and the accumulation of spatial and ecological knowledge at the group level. The Hadza’s diverse travel patterns create opportunities for frequent interactions where individuals can share ecological information that others are unaware of. By contrast, among Mpala baboons, the ‘ecological news’ gathered by one group member is largely equivalent to others’. The evolution of more Hadza-like patterns, we suggest, would generate conditions more conducive to communication about places and events beyond the here and now, a key adaptive feature of language.

The scenario we envision as conducive to the evolution of language involves central place foragers who travel widely and disperse during the day, and then aggregate afterwards. Under what conditions would it be beneficial for such individuals to share ecological information? We have not focused on that question here, but Bickerton [29] argued that a hominin dietary shift towards more hunting and scavenging would have generated new opportunities for mutualistic cooperation, and thus a context where individuals who could communicate with and recruit conspecifics for the defence, processing and carrying of large animal carcasses would benefit. Because hunter–gatherers cooperate in many foraging activities, the evolution of language to coordinate joint action for harvesting resources is a compelling idea [40–42]. Theoretically, one can imagine different temporal and spatial scales of dispersal and aggregation, different sorts of ecological features and adaptive challenges that may warrant cooperation and communication, and differences in the social composition of groups that would impact the costs and benefits of sharing news. This is all ripe territory for modelling, and agent-based models of social learning and information transmission are well suited to exploring these possibilities (e.g. Garg *et al*. [43]).

How did the highly diverse ranging patterns of hunter–gatherers arise? As noted by Marlowe [44], the evolution of technology—including sharpened sticks, pikes and spears would have improved hominin extractive foraging and hunting, predator defence and likely changed patterns of travel. The Hadza’s bows, arrows, knives, axes and digging sticks both make their hunting and gathering more effective and protect them from the same predators that afflict baboons. The development of such technologies is likely to have co-evolved with greater individual spatial autonomy and socio-spatial diversity, and thus, generated more contexts for the adaptive sharing of ecological information, recruitment and spatial coordination in general. A shift towards more technologically aided extractive foraging is likely to have also selected for larger brains [1]. Rosati [45] provides several examples of how foraging strategies predict cognitive traits of primates and calls for renewed attention to how diet, foraging strategies, and ranging behaviours might influence cognition. Rosati also notes that human hunter–gatherers appear exceptional in terms of their ranging and the degree to which socially acquired information (culture) is employed in foraging strategies.

Language and storytelling are human universals, and many ethnographic studies have documented storytelling in hunter–gatherer camps (e.g. [46]). Marlowe describes Hadza storytelling as both a form of entertainment and a way to convey vital information [37, p 66]. Scalise Sugiyama [47] argues that acquiring locally adapted foraging knowledge is a key function of narrative storytelling in hunter–gatherers. In her study of Ju/’hoansi storytelling, Wiessner [48] notes that economic plans are a frequent topic discussed during daylight hours and that events of the day are also retold by firelight at night. In their study of Agta storytelling, Smith *et al*. [49] note that narrative stories often convey messages that are important for coordinating behaviour and planning foraging activities. These observations align closely with our experiences in Hadza camps, where, regardless of one’s luck in foraging, everyone returns to camp with a story to share.

## 4. Material and method

All the code needed to reproduce this paper’s figures and text-reported results are shared via the Open Science Foundation (https://osf.io/c6gr5/ and doi: 10.17605/OSF.IO/C6GR5).

### (a) Hadza GPS data

**Figure 7.**
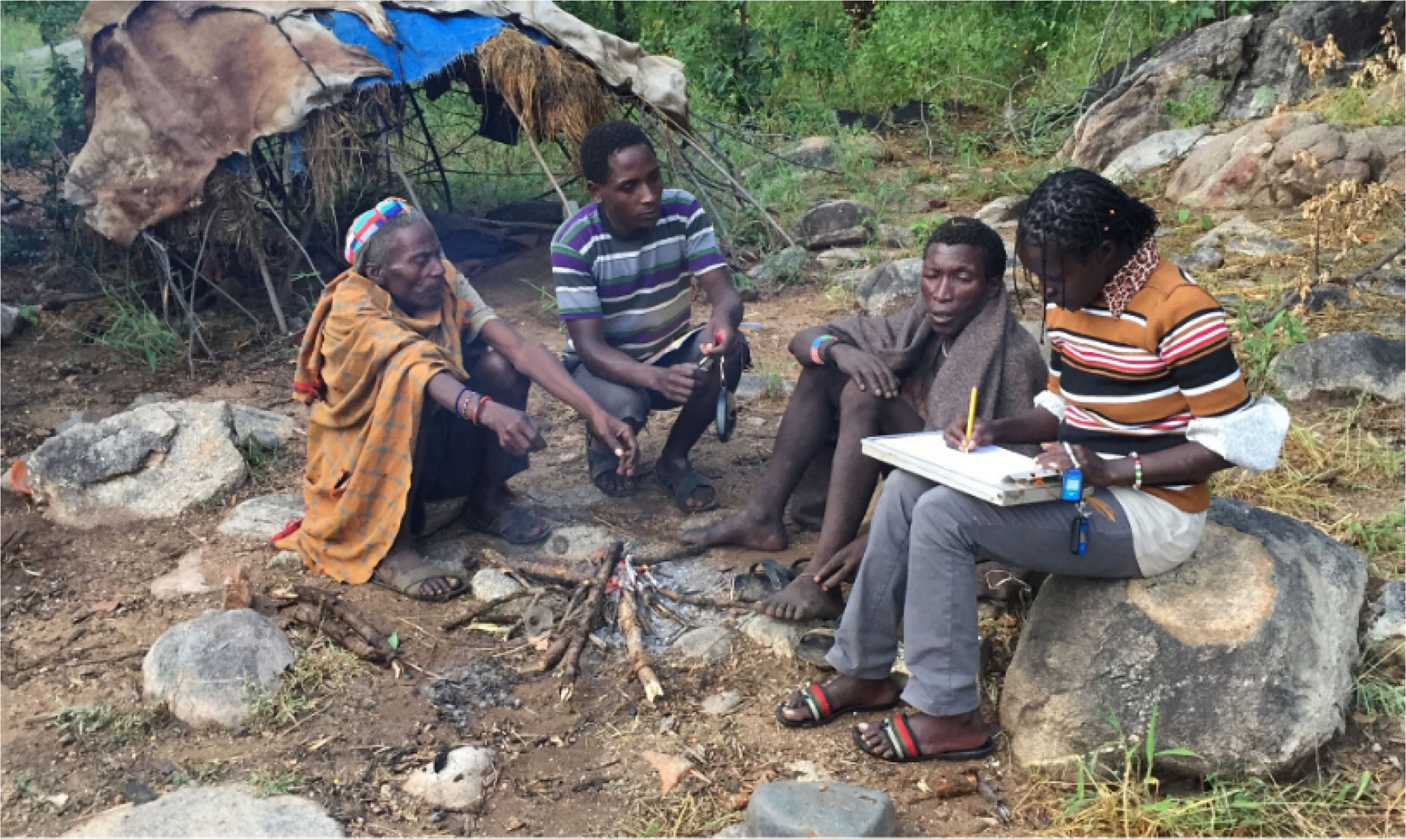
Co-authors BP and MA collecting GPS data using lightweight Canmore GPS devices.

Before participating in the study, all participants were informed about the research and gave their consent. This research received authorization from the Institutional Review Boards at Harvard University, Yale University, Hunter College, the University of Arizona and the University of California, Los Angeles, as well as from the Tanzania Commission for Science and Technology and the National Institute for Medical Research in Tanzania. GPS data were collected in 15 Hadza camps between 2005 and 2018 where the residents were subsisting from hunting and gathering for the vast majority of their diet (figure 7). The sample includes 2078 person-days of travel, and 179 participants (87 female, 92 male, mean age = 36). The GPS data record individuals’ locations every 5 s. On average, devices were put on at 07:46 and stopped recording at 18:58. Full details of the methods are shared in open-access publication Wood *et al*. [33].

### (b) Mpala olive baboon GPS data

The baboon GPS data were collected in August 2012 and shared in moveBank. The original raw data recorded individual locations once per second, and we have resampled the data to a resolution of one location per five seconds, in order to make the temporal resolution equivalent to the Hadza data. In the moveBank data, there is one individual who has spatial data but has no corresponding demographic data provided, which is individual 2459. This individual has been dropped from the analyses. Four other days of GPS data were excluded: the GPS data of individual 2452 on 14 August 2012 were excluded because the GPS readings were clearly in error, and the device only recorded locations for 9.5 h that day, while all other individuals had their locations recorded for 11.9 h. The locations of individual 2432 on 5 August 2012 were excluded because this track only recorded for 1.55 h. The locations of individual 2450 on 5 August 2012 were excluded because they only recorded positions for 4.57 h; the locations of individual 2433 on 6 August 2012 were excluded because they only recorded 7.1 h of data.

### (c) Inter-individual proximities

We calculated inter-individual proximities every 5 s on each day, for every possible dyad of individuals. Using this large dataset, we then made simple calculations of the mean, median and standard deviation in proximity across all times and dyads, within each study location. Data from each study location were then stratified by hour (e.g. hour 7 covers 7:00:00 until 7:59:59) and quantiles of dyad distances were calculated for each hour.

### (d) Dyadic measure of landscape visitation

The landscape visited by each individual on each day ($L_{i,d}$) was calculated from the GPS data using the function *rasterize* in the R package *raster* [50]. Using these raster representations, we then measured the similarity and difference in landscape areas visited each day for each dyad. For this calculation, each 10 m^2^ raster cell in the landscape was analysed for each day and for each dyad (*i, j*) and placed into one of four sets:

— a) Visited by neither *i* nor *j*.
— b) Visited only by *i*
— c) Visited only by *j*
— d) Visited by both *i* and *j*.

Counts of the raster cells in each of these sets were made (*n*_a_, *n*_b_, *n*_c_, *n*_d_), and a count of all cells visited by any member of the dyad (*n*_e_ = *n*_b_ + *n*_c_+ *n*_d_). The measure ‘fraction different’ represents the fraction of the visited landscape that was visited by only one member of the dyad, and is calculated as (*n*_b_+*n*_c_)/*n*_e_. The measure ‘fraction similar’ is calculated as *n*_d_/*n*_e_ and represents the fraction of the dyad-visited landscape that was visited by both members of the dyad.

### (e) Cumulative measures of landscape exploration

For each study community and each day, we assessed the relationship between the number of individuals included in the analysis and the cumulative size of the landscape visited by that number of considered individuals. Within each day, we generated a random order of individual IDs that dictated the order by which subjects’ GPS data would be entered into the analysis. For the first individual considered within a day, all the 10 m^2^ raster cells intersected by this individuals’ path of travel are included in the cumulative metric of total landscape visitation. For the second individual, we carried out a union of the first and second individuals' rasters of land visitation and then summed the number of raster cells in that union. We continued this process for each day until all the paths of travel for all individuals had been entered into the cumulative sum.

### (f) Sex differences in landscape use

For the Mpala baboon data, we identified all the 10 m^2^ raster cells intersected by all male tracks and all the cells intersected by all the female tracks. Set operations were then used to calculate the amount of visited landscape that was visited only by males, only by females or by both males and females. The same analyses were carried out for 12 different Hadza camps and the results across these samples averaged [33, table 1].

## Data Availability

All the code and data needed to reproduce the text-reported results and figures are available at the Open Science Foundation [51].
